# Editorial: Synchronization, Swarming and Emergent Behaviors in Complex Networks and Neuroscience

**DOI:** 10.3389/fncom.2022.846189

**Published:** 2022-03-07

**Authors:** Andrea Duggento, Spase Petkoski, Tomislav Stankovski, Nicola Toschi

**Affiliations:** ^1^Department of Biomedicine and Prevention, University of Rome Tor Vergata, Rome, Italy; ^2^Institut de Neurosciences des Systèmes, INS, INSERM, Aix Marseille Université, Marseille, France; ^3^Faculty of Medicine, Ss Cyril and Methodius University, Skopje, North Macedonia; ^4^Department of Physics, Lancaster University, Lancaster, United Kingdom; ^5^Department of Radiology, Athinoula A. Martinos Center for Biomedical Imaging, Boston, MA, United States

**Keywords:** synchronization, time-delayed coupling, swarmalators, brain dynamics, complex networks, effective connectivity, dynamical systems, emergent

A vast number of systems in nature display the peculiar ability to spontaneously enter synchrony across multiple spatial and temporal scales, and the study of natural synchronization has inspired numerous transdisciplinary applications in fields ranging from sensor networks and signal transmission to systems biology and physiological systems dynamics. While synchronization between sub-units has been vastly investigated as a function of local as well as coupling dynamics, the recent advent and exponential growth of complex network science has provided novel, fertile ground for deepening our insight into synchronization phenomena as a function of intrinsic, possibly dynamical network properties.

For example, the study of synchronization in hierarchical networks and, in particular, of how global dynamics can emerge from different network motifs and how mesoscale topology and time-delays due to propagation, as well as microscopic properties can influence whole-network synchronization, is beginning to provide solid stepping-stones for a better understanding of complex neuronal networks. In this context, recent neuroscience research has suggested that the synchronization of low-level elements in neural populations are instrumental to the dynamical emergence of higher-lever neural functional units, which in turn interact to generate and regulate complex behavioral patterns in health and disease. Recent simultaneous macro- (gross neural activities) and the micro- (single/multi-neuron activities) scale assessments are also providing evidence for complex, cross-scale brain interactions which are not yet well understood.

The study of synchronization in time-varying networks has very recently posited the existence of so-called “chimera-states”, whose appearance and disappearance in neuronal networks has been explained as an interplay of integration and segregation which gives rise to metastability. Similarly, the study of swarming in natural systems has very recently prompted ideas that exploit similarities with synchronization phenomena to define so-called “swarmalators”, i.e., units that are able to both swarm and synch, and are possibly governed by unifying physical principles such as energy conservation. Such systems exhibit rich spatiotemporal dynamics and may offer additional insight into mechanistic as well as statistical modeling for natural systems, as well as potential technological applications such as bio-computing and swarm robotics. In this context, two emergent phenomena in the hippocampus, i.e., self-stabilizing maps as well as temporal reorganization through sharing oscillatory dynamics, have provided explanations for decentralized self-organization and distributed communication in the brain.

Often the type of phenomena and the features that arise from the dynamics of the synchronized system are intrinsically pan-scale, and cover both ends of the spectrum of “biological networks,” from neuronal functioning to the collective behavior of individuals. For example, in their perspective paper Ribeiro et al. introduced and summarized the striking similarities between flocking in animal group dynamics and neuronal populations in the brain. The parallelism includes the key feature of scale-free correlation functions which (both in animal flocks and mammal brains) arise from the absence of a central control, and inherently lead to better response to external perturbation (e.g., intrusions by predators or external stimuli). The parallelism extends from interaction-length vs. correlation-length considerations, to effects due to structure heterogeneities. Another key feature is the nature of the phase transitions underlying the collective properties of animal groups and neuronal populations, which are at the basis of our understanding of phase-change mechanisms. Ribeiro et al. show that these two key features (near-critical dynamics and scale-free organization) provide maximal information transmission and key dynamic range advantages for both flocks and brains. Cross-frequency coupling is an important aspect of neural interactions, however, its origin is not yet fully understood. One paper Sinha et al. performed a theoretical study about the effect of the number of synaptic layers in descending pathways on the expression of cross-frequency coupling between supraspinal input and the cumulative output of the motoneuron pool using computer simulations of Hodgkin–Huxley like neuron models. They showed that the cross-frequency coupling is dominant in multi-synaptic indirect motor pathways, paving the way for a future human subject study.

This Research Topic also includes an *in-vitro* and *in-vivo* validation of how cross-frequency coupling and oscillatory synchrony give rise to complex communication strategies between clusters of neurons. Kawai explores the emergence of noise-causing stochastic resonance (reverberation) and coherence, resonance-like phenomena in neurons of the vagal complex by using brainstem preparations. Through these phenomena, which are rarely observed in *in-vitro* and *in-vivo*, this study demonstrates the role of neuronal noise with respect to the robustness and resilience of life-sustaining vagal functions.

Another *in-vivo* study França et al. provides evidence that beta2 frequency (20–30 Hz) oscillations in hippocampal activity are linked with novelty detection and processing. In particular, in behavioral task experiments in mice, animals are exposed to different degrees of novelty content. Simultaneous extracellular recordings of the CA1 hippocampal region and mid-prefrontal and posterior parietal cortex are analyzed to demonstrate that beta2 power increases with both spatial and object novelty content, and that novelty modulates oscillatory coherence in hippocampal-cortical circuitry.

Even though the topographic organization of biological networks is often known, their role in influencing the spatiotemporal dynamics of population activity is not understood, creating a theoretical gap between micro- and macro-scale observations. One paper Yu, Bouteiller et al., aims to fill this gap by studying the CA3 subfield of the hippocampus in rats (which has been investigated extensively from a topographical point of view) by providing detailed information about its connectivity.

Hippocampal structures (in humans and rats) are also investigated in Yu, Wu et al., with a two-fold approach that encompasses both human functional magnetic resonance and animal electrophysiology, in combination with molecular and biochemical evaluations. The authors demonstrate that chronic pelvic pain alters functional connectivity between the anterior cingulate cortex and hippocampal pathway.

Two contributions to this Research Topic analyzed neuronal masses that describe the mean-field activity of populations of theta neurons and quadratic integrate and fire (QIF) neurons. These are often referred to as next generation neural mass models (Coombes and Byrne, 2018), and they all stem from the exact mean-field formalism based on the Lorentzian ansatz, introduced in a seminal paper by Montbrió et al. (2015). Theta-nested gamma oscillations were investigated in two variants of neuronal mass of QIF neurons by Segneri et al.: the pyramidal interneuronal network gamma and the interneuronal network gamma. In both set-ups the system is driven with a sinusoidal theta-forcing in the proximity of a Hopf bifurcation, giving rise to the mixed theta-gamma rhythms that always display phase amplitude coupling. These types of mixed oscillations have been reported in many areas of the brain and they have been replicated through optogenetic theta frequency stimulation. In Lin et al. synaptic diversity was shown to suppress the complex collective behavior in networks of theta neurons. Aiming to account for more realistic heterogeneous inter-neuronal coupling strengths, the authors show (analytically) that the collective macroscopic behavior of a network of theta neurons gives rise to complex dynamics, but the increase of synaptic diversity leads to suppression of most of the dynamical structures, selecting simple collective equilibrium states in physiologically relevant regions.

Models of neuronal populations were also analyzed in Schumm et al. in the context of impairment of rhythms between microcircuits caused by neuronal degeneration after traumatic brain injury. The authors studied changes in the synchronization in networks of Izhikevich integrate-and-fire neurons, which also adapt according to spike-timing-dependent plasticity. The results indicate that inherent resilience strongly depends on the connectivity with highly synchronized circuits, which are largely protected against the effects of neuronal deletion.

Often the transition from synchronized to desynchronized states involves a so-called chimera or solitary state (a sort of coexistence of coherent and incoherent dynamical evolution). Kushwaha et al. employ large numerical simulations to investigate whether the creations of chimera and solitary states can be predicted from network topology and distributions of delays between oscillators of the network.

Qin et al. concentrate on ensemble spike events to extract the network evoked by acupuncture manipulations. In their theoretical analysis and numerical simulations they describe network response activities employing Bayesian theory, and estimates of network spike correlations along with *in-silico* experiments, which are used to define the relationship between spike correlations and synchronous spike events.

In Puxeddu et al. the authors conducted a comprehensive, extensive, and systematic comparative analysis among multilayer community detection methods. They looked at three different clustering approaches and four algorithms based on single-layer modularity, multilayer modularity, and evolutionary clustering. First, they analyzed the performance of the methods in ground truth networks. Their results suggested that the performance of the algorithms depends on the network features, such as the number of clusters, number of layers, and level of noise in the network. Their application to real EEG networks confirms the feasibility and usefulness of these methods.

Javed et al. studied the process of human aging. They used EEG-based scalp level analysis to identify aging-related alterations in synchronized brain regions. Starting from EEG data, the method decomposes the oscillations, calculates instantaneous features (amplitude and frequency), after which it extracts band-wise topographic maps. These topographic maps have shown the capability of capturing age-related changes in both spatial distribution and temporal characterization.

Racz et al. analyzed delta band (0.5–4 Hz) neural activity in schizophrenia. They used multifractal and entropy-based analysis to compare patients to controls, placing special attention on detecting the time-varying properties of neural interactions. Their results imply that the dynamic features of brain connectivity (e.g. multifractal properties and entropy) are potent markers of altered neural dynamics in schizophrenia.

Deschle et al. performed a theoretical study of how well the mass models resemble the real mean dynamics of a neural population. They tested the validity of neural mass models and whether the population under study comprised a mixture of excitatory and inhibitory neurons that are densely (inter-)connected. They found that these methods represent the mean dynamics for specific conditions, but not for all, and conclude that mass models should be used with great care.

In another theoretical contribution, Ghosh et al. employed Izhikevich neuron models to study the emergence of mixed mode oscillations. These are complex firing patterns that are neither spiking nor bursting activity alone. Instead, oscillations are distributed over different amplitudes, and the firings alternate between large and small amplitude oscillations. Ghosh et al. analyze mixed mode oscillations in a random and a small-world network of various neurons for different coupling strengths.

Spontaneous BOLD fMRI activity of the brain in the context of Resting State Networks (RSN) was analyzed by two studies. Amemiya et al. analyzed global vs. network-specific regulations as the source of intrinsic coactivations in the RSN. By using temporal independent component analysis, the authors investigated mechanisms that can give rise to network-specific coactivations. The time lag of global oscillations was shown to contribute to the RSN synchronization as much as the locally confined activities. The results thus confirm an equally important role of network-specific regulation for its coactivation, regardless of whether vascular artifacts contaminate the global component in fMRI measures.

Vohryzek et al. looked for recurrent excursions into functionally-relevant BOLD phase-locking states to adequately assess the biophysical mechanisms governing intrinsic brain activity. Clustering BOLD phase-locking patterns from 100 healthy participants from the Human Connectome Project into a set of k states. The authors demonstrate that the cluster centroids closely overlap with reference functional subsystems. In addition, the results reinforce the mechanistic scenario that RSN is the expression of erratic excursions from a baseline synchronous steady state into weakly-stable partially-synchronized states – i.e., ghost attractors.

In summary, with the advent of mass-scale exploitation of biologically inspired mechanisms as inspiration for artificial intelligence, there has been a surge in renewed interest in complex phenomena in biological networks. The need to understand the global functioning of complex neuronal networks has fueled theoretical and applied research of the classical topics of dynamical systems—such as studies of synchronization—to the now mature but ever-growing field of complex network science. While more and more observations and experiments in neuroscience investigate the functional macro-scale of network organization through a top-down approach, and system biology paradigms are creating an understanding of micro-scale complex phenomena, the physiological mechanisms of biological networks at the “meso-scale” are not yet well understood. This Research Topic collects contributions across theory, methods, and applications of complex networks to provide a reference point for the interested reader in this renewed and ever-increasing field.

## Author Contributions

All authors listed have made a substantial, direct, and intellectual contribution to the work and approved it for publication.

## Funding

SP received support from the European Unions Horizon 2020 research and innovation program under grant agreement No. 945539 (SGA3) Human Brain Project.

## Conflict of Interest

The authors declare that the research was conducted in the absence of any commercial or financial relationships that could be construed as a potential conflict of interest.

## Publisher's Note

All claims expressed in this article are solely those of the authors and do not necessarily represent those of their affiliated organizations, or those of the publisher, the editors and the reviewers. Any product that may be evaluated in this article, or claim that may be made by its manufacturer, is not guaranteed or endorsed by the publisher.
